# Shear Wave Elastography versus Strain Elastography in Diagnosing Parathyroid Adenomas

**DOI:** 10.1155/2020/3801902

**Published:** 2020-03-17

**Authors:** Laura Cotoi, Daniela Amzar, Ioan Sporea, Andreea Borlea, Dan Navolan, Flore Varcus, Dana Stoian

**Affiliations:** ^1^PhD School Department, “Victor Babes” University of Medicine and Pharmacy, Timisoara, Romania; ^2^Department of Endocrinology, “Victor Babes” University of Medicine and Pharmacy, Timisoara, Romania; ^3^Department of Gastroenterology and Hepatology, “Victor Babes” University of Medicine and Pharmacy, Timisoara, Romania; ^4^Department of Obstetrics and Gynecology III, “Victor Babes” University of Medicine and Pharmacy, Timisoara, Romania; ^5^Department of Surgery II, “Victor Babes” University of Medicine and Pharmacy, Timisoara, Romania

## Abstract

**Objectives:**

The aim of the study was to compare elastographic means in parathyroid adenomas, using shear wave elastography and strain elastography.

**Methods:**

This prospective study examined 20 consecutive patients diagnosed with primary hyperparathyroidism and parathyroid adenoma, confirmed by biochemical assay, technetium-99 sestamibi scintigraphy, and pathology report, after parathyroid surgery. All patients were examined on conventional 2B ultrasound, 2D shear wave elastography, and strain elastography. We determined using 2D shear wave elastography (SWE) the elasticity index (EI) in parathyroid adenoma, thyroid parenchyma, and surrounding muscle and examined using strain elastography the parathyroid adenoma, and determined the strain ratio with the thyroid tissue and muscle tissue.

**Results:**

All patients had positive sestamibi scintigraphy and underwent surgery, with confirmation of parathyroid adenoma in all cases. The mean parathormone (PTH) value before surgery was 153.29 pg/ml (36.5, 464.8) and serum calcium concentration was 10.5 mg/dl (9, 11.5). We compared using 2D-SWE and strain elastography parathyroid adenoma with thyroid tissue and with surrounding muscle. The mean EI measured by SWE in parathyroid adenoma was 4.74 ± 2.74 kPa and in thyroid parenchyma was 11.718 ± 4.206 kPa (mean difference = 6.978 kPa, *p* < 0.001), and the mean EI value in muscle tissue was 16.362 ± 3.829 kPa (mean difference = 11.622, *p* < 0.001). Using ROC analysis, we found that an EI below 7 kPa correctly identifies parathyroid tissue. We evaluated parathyroid adenomas using strain elastography by color mapping and strain ratio as a semiquantitative measurement; however, we could not find any statistical correlation comparing the strain ratio obtained from the parathyroid adenoma with the thyroid tissue (*p*=0.485).

**Conclusion:**

Ultrasound elastography is a helpful tool in identifying parathyroid adenomas. A cutoff value below 7 kPa can be used in 2D-SWE. Color maps in strain elastography without adding strain ratio can be used, parathyroid adenoma being identified as score 1 in the Rago criteria.

## 1. Introduction

Primary hyperparathyroidism (PHPT) is the third most frequent endocrinopathy, after type 2 diabetes mellitus and thyroid disease. It is most commonly caused by an overactive parathyroid gland resulting in high serum parathormone (PTH) concentrations and consequent high serum calcium concentrations [1–7]. PHPT is nowadays commonly asymptomatic, with high prevalence among postmenopausal women (female–male ratio 3–4 : 1) [8–11], routine serum calcium evaluation contributing to an increase in disease diagnosis [6].

The most common PHPT cause is unique parathyroid adenoma [12]. Sporadic parathyroid adenomas represent 85–90% of cases [3], a smaller percentage is accounted for multiglandular disease and less than 1% is due to parathyroid carcinoma [2–4].

Active screening has increased the incidence of PHPT, as European studies have shown [5, 13]. A Swedish study showed that 3% of women and 0.7% of men over the age of 60 will present PHPT [14]. A prevalence study of PHPT conducted in Denmark evaluated that over a period of 11 years, there was an annual rate of 16 per 100.000 cases [15].

Primary hyperparathyroidism can be surgically treated, requiring accurate preoperative localization of the adenoma [16]. Most available preoperative localization techniques for PHPT are ultrasonography (US) and technitium-99 m sestamibi scintigraphy scan [17]. US is widely available, noninvasive, cheap, and repeatable [18, 19], and it can be used for positive diagnosis and also for therapeutic interventions (fine-needle aspiration and percutaneous interventions–ethanol, radio frequency, or laser ablation) [20]. Additional applications of US such as color and power Doppler, 3D imaging, contrast-enhanced ultrasonography, and elastography have increased the diagnostic value of ultrasound in diagnosing hyperparathyroidism [18], by decreasing the false-positive cases.

Important literature studies have concluded that the diagnostic value of US in parathyroid adenomas has a sensitivity of up to 76% and a specificity of 93.2% [21].

Elastography is a relatively novel technique, complementary to conventional ultrasonography. It can be used to assess tissue stiffness [22] with the potential of differentiating benign from malignant tissues [23].

Complementary to conventional ultrasound, elastography can be used to assess quantitative and qualitative information about tissue stiffness and can make the correlation between healthy tissue and pathological tissue. By measuring tissue elasticity, elastography has been validated as a marker of pathological states in many clinical areas [24], by improving diagnostic methods and establishing its role in breast tumours characterization [25], thyroid nodules [22], testicular cancer [26, 27], and liver pathology [28]. There are also studies conducted on primary and secondary hyperparathyroidism [29–32].

Elastography's efficacy in parathyroid disease is less studied, the major impediment being the lack of visualisation of normal parathyroid glands on conventional ultrasound. Elastographic measurements are not able to compare normal parathyroid tissue with hypertrophic or hyperplastic parathyroid lesions, thus using surrogate markers, such as thyroid tissue or sternocleidomastoid muscle.

### 1.1. Principle of Elastography

Elastography estimates tissue stiffness by applying compression force and measuring the distortion degree of the tissue [33]. Tissue strain is estimated by finite difference estimation of echo time-delays obtained cross-correlating processing before and after compression echo signals [34].

The distortion degree can be obtained using two methods, applying external pressure manually or via ultrasound transducer [33]. Shear waves are generated by short-duration acoustic beam or by converged ultrasound beams. They generate shear waves that diffuse transversally through the tissue.

Qualitative information are acquired through color maps, semiquantitative information through strain ratio (SR), and qualitative information through measurements (numerical values) [22].

#### 1.1.1. Strain Elastography

Real-time elastography (RTE) is an elastographic technique that requires external pressure in order to produce deformation of subjacent tissue. After applying a controlled external pressure, elastographic map is added on grey-scale conventional ultrasound mode and displayed as color maps (red for liquids, green for soft tissue, and blue for hard tissue), giving the physician qualitative information about the tissue stiffness [22].

Semiquantitative information can be obtained using strain elastography, by comparing tissue strain in the region of interest (ROI) of targeted tissue with another adjacent healthy tissue, computing a SR.

#### 1.1.2. Shear Wave Elastography

SWE assesses tissue stiffness by evaluating shear wave attenuation. Shear waves are induced vibrations by acoustic radiation force through a focused ultrasound beam, making it a more operator-independent technique [22, 35, 36]. Analyzing with ultrafast ultrasound acquisition sequence the particle displacements, quantitative measurements of tissue elasticity can be obtained [35].

Two elastographic methods use SWE: supersonic shear wave and acoustic radiation force impulse (ARFI) [37].

In supersonic shear wave, focused ultrasound beam is used and particle displacements of tissue can be measured with wave velocity (m/s) or by direct measurement in the ROI (kilopascals-kPa), acquiring quantitative data. Qualitative data are also available through color maps. In comparison to strain elastography, supersonic software displays a different color code—soft tissues are blue and stiff tissues are red [22, 38].

## 2. Objective

The objective of this prospective study was to determine, using SWE and RTE, the characteristics of parathyroid adenomas and to determine whether the techniques add information in the preoperative diagnosis of primary hyperparathyroidism cases.

## 3. Materials and Methods

This prospective study evaluated 20 consecutive patients diagnosed with primary hyperparathyroidism, from October 2018 to June 2019. All cases were patients aged over 18 years who presented solitary parathyroid adenoma, confirmed by biochemical evaluation, localization with technetium sestamibi scintigraphy (MIBI) and certified by pathology report after surgery (parathyroid adenoma excision). The study was approved by the Ethics Committee of our hospital and all patients signed a written informed consent. The study was in accordance with the Ethics Code of the World Medical Association.

### 3.1. Inclusion Criteria

All patients were adults with confirmed primary hyperparathyroidism. After performing all measurements, to conclude if there are any differences between patients with primary hyperparathyroidism and consequent autoimmune thyroid disease, we divided our study group into 2 subgroups. One group of patients with parathyroid adenoma and thyroid autoimmune disease (11 patients) and another group of patients with parathyroid adenoma, but without thyroid disease (9 patients). Only patients who underwent surgery were considered in the final analysis, with pathology report of parathyroid adenoma being the golden standard for diagnosis.

### 3.2. Exclusion Criteria

Ectopic parathyroid adenoma diagnosed by means of MIBI scintigraphy was excluded. Secondary cases of hyperparathyroidism were also not considered in the evaluation, regardless of whether the etiology was renal or digestive.

### 3.3. Conventional Ultrasound

Conventional B-mode parathyroid ultrasound was performed in all cases on two different ultrasound systems, with an Aixplorer system (SuperSonic Imagine, France) using a high-resolution linear transducer of 15–4 MHz and with Hitachi Preirus (Hitachi Medical Corporation, Tokyo, Japan) machine with a 6- to 13-MHz linear probe. Using grey-scale US, we evaluated the following parameters: thyroid dimensions (two dimensions in transverse scan and one dimension in longitudinal), thyroid volume, parathyroid adenoma dimensions, parathyroid adenoma volume, shape, and echogenity. Doppler US was performed in order to observe the presence of the peripheral vascular rim as a landmark sign for parathyroid adenomas. All patients were clinically and ultrasonographically evaluated by two practitioners, one with over a 15-year experience in thyroid, parathyroid, and neck ultrasound.

### 3.4. Shear Wave Elastography

After performing conventional ultrasound, 2D-SWE was performed with an Aixplorer system (SuperSonic Imagine, France) using a high-resolution multifrequency linear transducer of 15–4 MHz. Patients remained in the supine position, with hyperextension of the neck; the examiner maintained precise adherence of the probe to the examined area, without applying any manual compression, permitting the transducer to automatically induce vibrations in the tissue and avoiding any movements while waiting for image stabilization.

**Figure 1:**
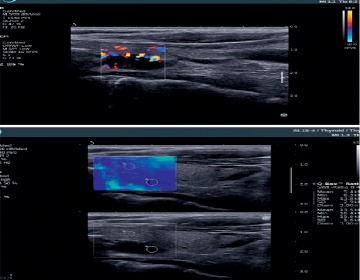
US and 2D-SWE evaluation of parathyroid adenoma.

Qualitative assessment was first obtained using real-time elastograms. Quantitative information was later obtained using a special software program. We determined the parameters identified as the elasticity index (EI). Default settings for parathyroid 2D-SWE evaluation are not established, and for the study, we used 2D-SWE thyroid settings (range 0–100 kPa). In SWE, quantitative measurements for determining tissue elasticity properties can be obtained within a ROI. On the Aixplorer system, the ROI is quantified as quantification box (Q-Box) (Figure 1). Q-Box is available only on frozen image and provides quantitative information about tissue stiffness. The summary of tissue elasticity properties is automatically displayed using machine software: mean stiffness value (SWE-mean), maximum stiffness value (SWE-max), minimum stiffness value (SWE-min), standard deviation (SWE-SD), and the diameter of the ROI. A ratio between two different areas of interest on the same image can be obtained using Q-Box ratio. The numerical parameter obtained—SWE ratio—allows the practitioner to compare the stiffness of two areas on the same image [7].

**Figure 2:**
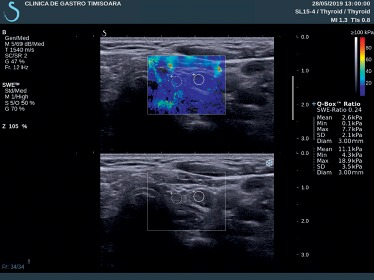
Q-Box ratio of parathyroid adenoma with thyroid tissue.

We compared the EI using the Q-Box ratio of parathyroid adenomas with thyroid tissue and also with sternocleidomastoid muscle tissue (Figure 2). For each parathyroid adenoma, we performed five measurements and compared them with the measurements obtained from the thyroid parenchyma and surrounding muscle tissue.

### 3.5. Strain Elastography

RTE was performed after conventional ultrasound evaluation using a Hitachi Preirus (Hitachi Medical Corporation, Tokyo, Japan) machine with a 6- to 13-MHz linear probe.

**Figure 3:**
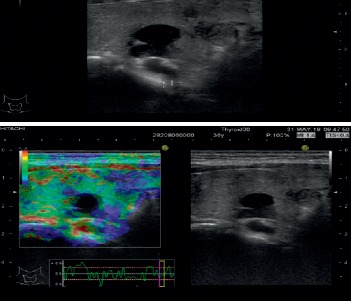
US and strain elastography of parathyroid adenoma.

Strain elastography depends upon externally induced deformation on the tissue. The stiff tissue has a lower degree of deformation and movement, compared with normal (elastic) tissue. Color elastograms (red—soft tissue to blue—stiff tissue) are displayed along with 2B-mode conventional ultrasound, giving qualitative information about tissue elasticity [22] (Figure 3).

Rago criteria [22, 39] used for thyroid nodular pathology was applied to the qualitative strain elastography evaluation: score 1—elasticity in the whole lesion, score 2—mostly soft, score 3—soft in the peripheral part of the lesion, score 4—the entire lesion is stiff, and score 5—stiffness that extends beyond the lesion's margins, infiltration in the surrounding tissue.

**Figure 4:**
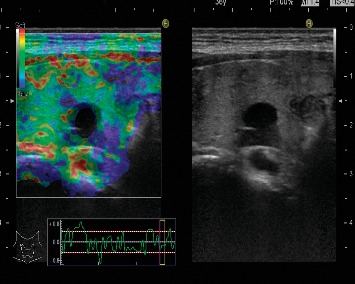
Color map on strain elastography with Hitachi machine. Score 1 on Rago criteria for qualitative strain elastography images.

The main limitation of this qualitative analysis is the depth of the lesions, giving incomplete colored maps. Most of the parathyroid adenomas evaluated were score 1 according to the Rago criteria (Figure 4).

Semiquantitative information can also be obtained using strain elastography, by comparing tissue strain in the ROI of the parathyroid adenoma parenchyma to the thyroid tissue or muscle. The result is an automatic SR.

We performed semiquantitative measurement (SR) for parathyroid adenoma versus thyroid parenchyma and, respectively, parathyroid adenoma versus sternocleidomastoid muscle.

### 3.6. Statistical Analysis

Data were collected and analyzed using SPSS v.25 (Statistical Package for the Social Sciences, Chicago, IL, USA). Continuous variables were presented as mean and standard deviation (SD), and categorical variables were presented as frequency and percentages.

We performed descriptive and inferential statistics analysis to summarize the characteristics of the study population. To compare patients with or without autoimmune thyroiditis, specific group-pairs with each other, we used the *t* test. For comparing the three measurements and to assess the EI (thyroid, parathyroid, and muscle), we used the ANOVA test, followed by a post hoc analysis with Tukey's HSD test.

The receiver operating characteristic (ROC) curve was employed to illustrate the diagnostic ability and the thresholds to discriminate between the parathyroid, and other structures (thyroid and muscle) were determined with the Youden index.

A *p* value of <0.05 was considered to indicate a statistically significant difference.

## 4. Results

We evaluated 20 patients (male to female ratio 1/19) with a mean age of 57.3 ± 13.33 years, mostly postmenopausal women, with confirmed primary hyperparathyroidism. Conventional ultrasound examination and both 2D-SWE and strain elastography were performed. Baseline characteristics of the study group are presented in Table 1.

Anatomical location of parathyroid adenoma can be a bias when using ultrasound for preoperative localization of the adenoma. In our study, we excluded ectopic localization of the parathyroid gland. Parathyroid adenomas in patients from our study were mostly found near the right superior thyroid lobe (9 adenomas), 3 were located near the right inferior thyroid lobe, 3 were located near the left superior lobe, and 5 near the left inferior lobe. The localization on ultrasound, compared with localization on scintigraphy and intraoperative findings, are depicted in Table 2.

**Figure 5:**
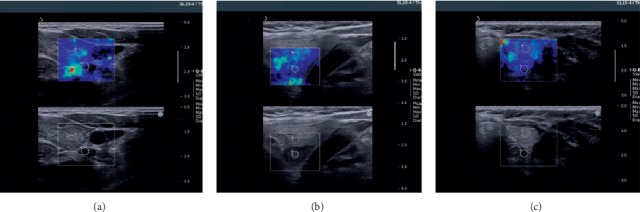
Image (a) –ultrasound appearance of hypoechoic cystic nodule; (b) –ultrasound appearance of hypoechoic homogeneously solid nodule; (c) –ultrasound appearance of mixed nodule.

On ultrasound findings, the mean parathyroid adenoma dimensions were 0.776 ± 0.50 cm, the maximum size found on ultrasound was 2.46 cm, and the minimum dimension was 0.34 mm. We found 13 parathyroid adenomas with cystic appearance (65%), 5 parathyroid adenomas with homogeneously solid and hypoechoic appearance (25%), and 2 adenomas had a mixed appearance (10%)—mostly cystic and one with an elongated shape (Figure 5).

### 4.1. 2B-SWE Results

In total, 20 patients were evaluated using 2D-SWE, and five measurements were made for each comparison. We noted our SWE result in Table 3, considering the minimum SWE value, the maximum SWE value, and the mean SWE value for each analyzed tissue.

**Figure 6:**
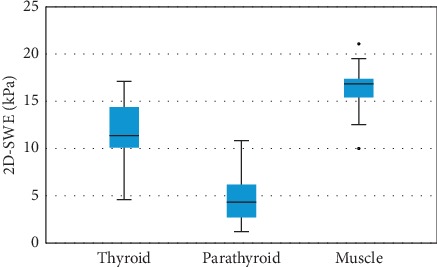
Comparison of mean SWE between parathyroid, thyroid, and muscle.

One-way ANOVA test demonstrated a statistically significant difference between parathyroid elasticity (kPa) and thyroid and muscle elasticity, when measured by 2D-SWE (*p* < 0.001) (Figure 6). Post hoc analysis with *T* test was conducted and a Bonferroni correction was applied, resulting in a significance level at *p* < 0.008, with higher parathyroid elasticity (4.74 ± 2.745 kPa) compared with normal thyroid tissue (11.718 ± 4.206 kPa), and respectively surrounding muscles (16.362 ± 3.829 kPa). The difference is significant when comparing SWE mean values of parathyroid adenomatous tissue with thyroid parenchyma (mean difference = −6.978 kPa, *p* < 0.001), respectively with mean SWE muscle values (mean difference = 11.622, *p* < 0.001).

The results summarizing AUROC, sensitivity, and specificity for each quantitative parameter are presented in Table 4. The best sensitivity and specificity are observed for SWE-mean value. By analyzing the parameters given by the ultrasound machine, we have found that the best accuracy is by analyzing the SWE-mean value, with a value of 92.5% for parathyroid/thyroid couple and 97.5% for parathyroid/muscle couple, followed by the SWE-min value with an accuracy of 90.0%, respectively 97.5%. SWE-min and SWE-max values could also be useful tools whenever borderline values are found, helping to discriminate structures and positively identify parathyroid adenomas.

**Figure 7:**
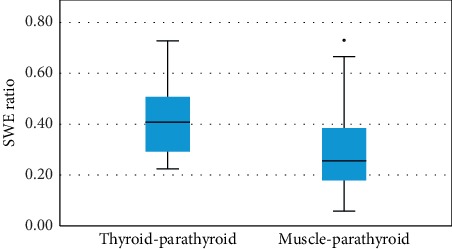
Values of SWE ratio of parathyroid versus thyroid elasticity, respectively parathyroid versus muscle elasticity.

We observed significant difference between the mean SWE ratio of parathyroid/thyroid compared with parathyroid/muscle SWE ratio (*p*=0.047) (Figure 7).

Evaluating the impact of parathyroid adenoma location on SWE measurement, we obtained the following results: anterior adenomas: mean SWE = 4.74 ± 2.75 kPa, SWE-max = 11.45 ± 6.19 kPa, and SWE-min = 1 ± 1.74 kPa; posterior adenomas: mean SWE = 4.66 ± 2.88 kPa, SWE-max = 11.62 ± 6.94 kPa, and SWE-min = 0.80 ± 1.42 kPa. We did not find significant differences related to the location of the parathyroid adenomas.

There were no differences in SWE ratio of parathyroid adenoma, compared to healthy thyroid tissue (9/20 cases), mean SWE ratio = 0.418 ± 0.230, and the ratio of parathyroid adenoma compared to autoimmune thyroid tissue (11/20 cases), mean SWE ratio = 0.416 ± 0.186, *p*=0.570.

Even if there are significant differences when using SWE ratio to determine the parathyroid adenomatous tissue, the SWE ratio values—parathyroid/thyroid and parathyroid/muscle, are similar to one another, and there is no clear delimitation between the two reports. Because of the contiguity of the SWE ratio values, we cannot determine the best SWE ratio to use. The most reliable method to differentiate the parathyroid tissue from surrounding tissues is by using the mean SWE value.

### 4.2. Strain Elastography Results

The initial qualitative analysis found 15 of 20 cases with score 1 on color map evaluation, according to the Rago criteria. Two cases had blue/green/red (BGR) sign due to lack of wave transmission.

Data obtained through the semiquantitative analysis using the strain elastography technique are summarized in Table 5. Using the *T* test, we found no significant differences in the SR value of parathyroid versus thyroid tissue compared with the SR value of parathyroid versus muscle tissue (*p*=0.485). Evaluation was possible in 18 cases. Two cases had a negative scan due to the depth of the parathyroid lesion.

There were also no differences in the SR of parathyroid adenoma versus healthy thyroid tissue (9/20 cases), mean SR = 1.465 ± 1.458, and the SR of parathyroid adenoma versus autoimmune thyroid disease tissue (11/20 cases), mean SR = 1.656 ± 1.746, *p* = 0.481.

### 4.3. Comparing SWE and RTE

**Figure 8:**
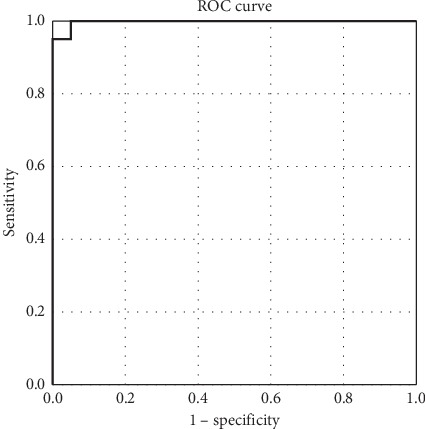
AUC for prediction of parathyroid adenoma using mean SWE for parathyroid (muscle as reference tissue).

With histopathological results of the parathyroid adenomas as gold standard, ROC curve was used to evaluate the discriminative power of elastography US examination. Comparing the mean SWE value of parathyroid with the mean SWE value of surrounding muscle, the area under the curve (AUC) was 0.997 (95% CI [0.990; 0.999]) (Figure 8). A value over 10.47 kPa has a specificity of 95% and a sensitivity of 100% in identifying muscle tissue, using parathyroid adenoma as reference.

**Figure 9:**
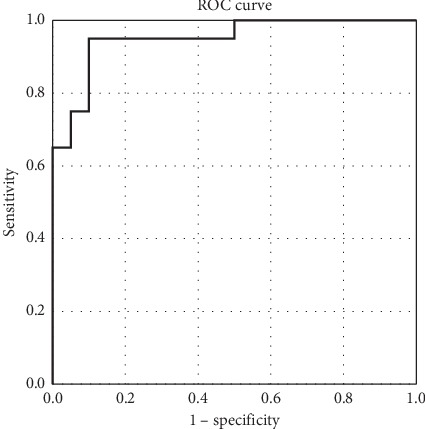
AUC for prediction of parathyroid adenoma using mean SWE for parathyroid (thyroid tissue as reference).

The mean SWE of parathyroid adenoma was compared to the mean SWE of thyroid tissue, regardless of whether the patient presents with an autoimmune thyroid disease, and the ROC curve obtained after evaluating the parameters, with AUC of 0.950 (95% CI [0.886; 0.999]) (Figure 9). A value over 7.28 kPa has a specificity of 90% and sensitivity of 95% in differentiating thyroid parenchyma, using parathyroid adenomatous tissue as reference.

**Figure 10:**
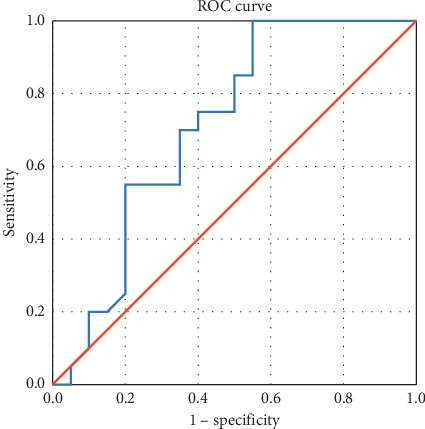
AUC for prediction of parathyroid adenoma using SWE (SWE ratio parathyroid/thyroid).

**Figure 11:**
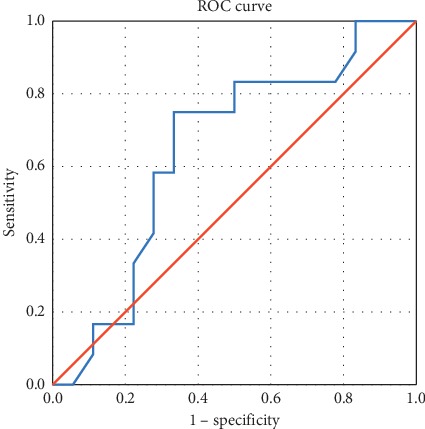
AUC for prediction of parathyroid adenoma using strain elastography (SR parathyroid/thyroid).

When comparing the two elastographic methods for identifying parathyroid adenomas, we compared the two ROC curves. The SWE method has a higher sensitivity and specificity, if parathyroid adenoma is compared with thyroid tissue (regardless of the presence of autoimmune thyroid disease) or surrounding muscle–AUC curve 0.70 (95% CI [0.544; 0.876]) (Figure 10), compared with the specificity and sensitivity of strain elastography–AUC curve 0.646 (95% CI [0.442; 0.850]) (Figure 11). The optimal point on SWE ratio parathyroid/thyroid can be set at 0.3030 with a sensitivity of 75% and a specificity of 60%.

The same point was set for RTE elastography using SR, with a point set at 1.4 using the Youden index, with a sensitivity of 75% and a specificity of 66.7%.

## 5. Discussion

In this prospective study, we compared elastographic features, both in SWE and strain elastography of confirmed parathyroid adenomas with surrounding thyroid tissue and sternocleidomastoid muscle. The study included only solitary parathyroid adenomas that were initially localized in conventional B-mode ultrasonography. Our study included mostly female patients, empowering the fact that single parathyroid adenomas are more prevalent among postmenopausal women [14].

The role of elastographic measurements in the parathyroid field is advancing, as several studies on the elasticity of parathyroid adenomas have been conducted and have shown that elastography is a helpful technique in differentiating parathyroid pathology [19, 29–31, 40–42].

In our study, tissue stiffness of parathyroid adenoma was best assessed by measuring mean SWE, providing quantitative information that may be useful in localizing parathyroid adenomas. The mean (±SD) SWE value assessed was 4.74 ± 2.745 kPa for all parathyroid adenomas enrolled in the study. Compared with thyroid parenchyma (mean SWE value 11.718 ± 4.206 kPa) and surrounding muscle (mean SWE value 16.362 ± 3.829 kPa), the elasticity of parathyroid adenoma was significantly lower than both thyroid and muscle tissue. Taking into account that our study included a limited number of patients, we evaluated using cross-tabulation on ROC curves that the best mean SWE cut-off value for predicting parathyroid adenomas should be below 7 kPa.

Polat et al. [19] examined parathyroid lesions using the SWE virtual touch imaging quantification (VTIQ) method and compared the shear wave velocity (SWV) of parathyroid lesions with inflammatory cervical lymph nodes. The mean SWV of parathyroid adenomas (2.16 ± 0.33 m/s) was higher than the mean SWV of parathyroid hyperplasia (1.75 ± 0.28 m/s) and higher than that of cervical lymph nodes (1.86 ± 0.37 m/s). They established a cut-off value greater than 1.92 m/s for diagnosing parathyroid adenoma.

Azizi et al. [29] compared parathyroid adenomas using SWE VTIQ with thyroid tissue. They found that parathyroid adenomas have a lower SWV mean of 2.01 m/s (±0.24) than the normal thyroid tissue of 2.77 m/s.

Batur et al. [40] measured the elasticity of parathyroid adenomas using ARFI imaging 2D-SWE and compared it to benign and malignant thyroid pathology. The reported results showed that parathyroid adenomas (mean SWV value of 3.09 ± 0.75 m/s) have a lower elasticity than benign thyroid nodules (2.20 ± 0.39 m/s) and a higher elasticity than malignant thyroid lesions (3.59 ± 0.43 m/s).

Stangierski et al. [41] measured using the 2D-SWE elastographic method parathyroid adenomas and compared elasticity with benign thyroid nodules. Parathyroid adenomas had a mean SWE value of 5.2 ± 7.2 kPa, which was significantly lower than that of thyroid nodules of 24.3 ± 33.8 kPa, showing that parathyroid adenomas have a significantly lower EI than benign thyroid nodules.

The difference between parathyroid and thyroid parenchyma elasticity was significant, regardless of whether we compared parathyroid adenomatous tissue with healthy thyroid tissue or with autoimmune thyroid tissue. On ultrasound examination, most lesions were cystic (nine nodules), and we also found solid and mixed adenomas (five and two nodules), but we did not find any significant differences on elastography examination regarding the EI, both on shear wave and on strain elastography. Also, the location of the parathyroid adenoma had no influence on the elastographic measurements.

Thyroid nodules are extremely common in the general population, up to 5% prevalence on palpation and over 50% discovered in autopsies [43]. Elastography has found its place in differentiating thyroid nodules, with a general consensus in major guidelines [44–46] and important papers in the field [47–49]. Thyroid nodules frequently pose a dilemma to the clinician, first in respect to the nature of the nodule and also when faced with patients with parathyroid adenomas. In our study, we did not compare parathyroid adenoma elasticity with thyroid nodule elasticity because of the small number of thyroid nodules present in examined patients.

Literature studies conducted on thyroid nodules on SWE have found EI values of 150 ± 95 kPa [38], 30.7 kPa [50], and 115 ± 60.4 kPa [51] for malignant nodules, and values of 36 ± 30 kPa [38], 18.7 kPa [50], and 41 ± 25.8 kPa [51] for benign nodules. EI values reported for normal thyroid tissue were 15.9 ± 7.6 kPa [38], 13.6 kPa [50]. Therefore, the EI index of malignant and also benign nodules is higher than the EI of normal thyroid tissue; thus, we could conclude that the EI of parathyroid adenoma should be lower than the EI of both normal thyroid tissue and thyroid nodules.

We also evaluated patients using strain elastography, resulting in qualitative and semiquantitative data. We found a mean SR of 1.46 for parathyroid/thyroid and 1.8 ± 0.9 for parathyroid/muscle and no significant statistic difference in SR between the two groups.

Because autoimmune thyroid disease does alter the elasticity of the thyroid tissue [52, 53], we compared the SR for the two subgroups formed–normal thyroid and chronic autoimmune thyroiditis, and did not find any significant differences between the two groups. We did not find any articles addressing this particular aspect.

RTE can be a useful tool in differentiating thyroid nodules [54]. RTE studies have found different SR cut-off values ranging from 2 to 4 [55], with lower values for benign nodules and higher values for malignant nodules [49]. We did not compare the SR of parathyroid adenoma and thyroid nodules.

Literature reports have studied and compared the elasticity of parathyroid pathology using strain elastography. In the first study conducted on 72 patients by Ünlütürk et al. [56], they found that parathyroid adenomas appear as stiff lesions (median SR = 3.56) and parathyroid hyperplasia have a lower stiffness and a higher elasticity score (median SR = 1.49). Isidori et al. [42] evaluated 79 patients with parathyroid disorders on Elastoscan Core Index (ECI) and compared parathyroid elasticity with lymph node and thyroid nodule elasticity. They found that ECI was significantly higher in malignant lesions than in benign lesions. Combining the ECI with conventional US, and in particular with shape and vascularization, can improve the differentiation of parathyroid lesions from lymph nodes and thyroid nodules.

We observed that 2D-SWE evaluation had better results in identifying parathyroid adenomas. The best diagnosis performance is observed using mean SWE parathyroid/muscle (AUROC = 0.997, Sn = 100%, Sp = 95%), followed by mean SWE parathyroid/thyroid (AUROC = 0.950, Sn = 95% Sp = 90%). RTE has lower diagnosis performance (AUROC = 0.646, Sn = 75%, Sp = 66.7%). To our best knowledge, this is the first study that used two major elastographic techniques to investigate parathyroid lesions and compared the accuracy of the methods, in order to determine the best method to localize parathyroid adenomas. The majority of literature studies had a small series of cases comparing parathyroid adenomas with normal thyroid tissue—57 patients [29], 36 patients [30], 21 patients [40], and 65 patients [41], or lymph nodes—66 patients [19]. Our study had a limited number of patients because of the small amount of time between diagnosis and surgery. We compared two important elastographic methods, in an effort to determine the most valuable method of determining the presence of parathyroid adenomas and differentiating parathyroid adenomas from surrounding tissue. We found that the best accuracy is obtained by analyzing the mean SWE—92.5%, followed by SWE-min—90% when compared with the thyroid tissue. When finding borderline values of mean SWE, SWE-min, SWE-max, and SWE ratio values could be useful tools to positively identify the parathyroid adenoma.

The study had certain limitations. First is the small number of patients enrolled in the study. In spite of the small number of patients, all patients underwent surgery, thus confirming by histopathological examination of the parathyroid lesions. The depth of parathyroid adenomas was also a limitation; it did not allow positive results on strain elastography scans in all cases. Based on the elastography studies in the literature [29] and the current study on parathyroid pathology, we could consider elastography as an useful tool in correctly identifying parathyroid adenoma, considering also the different tissue composition, vascularity pattern, and subsequent tissue stiffness in comparison to surrounding tissue. There are clinical implications regarding the use of elastography in diagnosing primary hyperparathyroidism. It is a simple, noninvasive, operator-independent, repeatable, and reproducible method, which can be used complementary to conventional ultrasound and scintigraphy examination, distinguishing between parathyroid adenoma, and thyroid and muscle tissue. It could furnish relevant information regarding parathyroid elasticity and could be a useful tool in localizing parathyroid adenomas in patients with primary hyperparathyroidism.

## 6. Conclusion

To conclude, the aim of this prospective study was to quantify the value of strain elastography and 2D-SWE in localizing parathyroid pathology. Although strain elastography can be a useful qualitative tool by using color mapping, 2D-SWE can offer a better differentiation on tissue elasticity when diagnosing parathyroid adenomas. By using this elastographic technique, a value less than 7 kPa for mean EI is suggestive for parathyroid adenoma. Also, when using the SWE ratio, a value less than 0.3030 when comparing with thyroid tissue, regardless of whether the patient is healthy or with autoimmune disease, is highly suggestive for parathyroid adenomas.

## Figures and Tables

**Table 1:** Baseline characteristics of the study.

Characteristic	Study group
F/M	19/1
Age (years)	57.3 ± 13.33
Adenoma volume (ml)	0.21 ± 0.32
Parathormone (pg/ml)	153.29 ± 118.43
Ionized serum calcium (mmol/l)	1.23 ± 0.2
Total serum calcium (mg/dl)	10.5 ± 0.96
Urinary calcium (mg/24 h)	225.89 ± 105.33
Urinary phosphorus (g/24 h)	0.74 ± 0.16
Diuresis (ml)	1966.5 ± 393.73
Glomerular filtration rate (ml/min/1.73 m)	104.74 ± 17.42
25-OH vitamin D (ng/ml)	23.07 ± 8.77
TSH (*μ*U/ml)	3.32 ± 4.05
FT4 (pmol/ml)	15.52 ± 2.92
Presence of autoimmune thyroid disease	11 (55%)
Thyroid volume (ml)	11.24 ± 5.37
Presence of osteoporosis	10 (50%)
T-score spine	−1.43 ± 1.32
T-score hip	−1.72 ± 1.18
Bone density mass (spine)	0.95 ± 0.21
Bone density mass (hip)	0.77 ± 0.17

F, female; M, male.

**Table 2:** Location on ultrasound, scintigraphy, and intraoperative findings of parathyroid adenoma.

Parathyroid adenoma location	Ultrasound	Scintigraphy	Intraoperative findings
Right superior lobe	9	7	8
Right inferior lobe	3	5	4
Left superior lobe	3	2	3
Left inferior lobe	5	6	5

**Table 3:** 2D-SWE results: mean value ± SD.

	Mean SWE (kPa)	Min SWE (kPa)	Max SWE (kPa)
Parathyroid adenoma	4.74 ± 2.745	1 ± 0.434	11 ± 6.266
Thyroid tissue	11.718 ± 4.206	7.013 ± 4.532	18.133 ± 7.789
Muscle tissue	16.362 ± 3.829	11.412 ± 4.097	25.518 ± 7.933

**Table 4:** Sensitivity, specificity, and AUROC for measured SWE-min, SWE-max, and SWE-mean.

	SWE-min PTX/T	SWE-mean PTX/T	SWE-max PTX/T	SWE-min PTX/M	SWE-mean PTX/M	SWE-max PTX/M
Area under curve (AUC) value	0.957	0.950	0.765	0.998	0.997	0.955
Specificity	85%	90.0%	70.0%	95.0%	95.0%	80.0%
Sensitivity	95%	95.0%	80.0%	100%	100%	100%
PPV	86.4%	*90.5*%	72.7%	95.2%	*95.2*%	83.3%
NPV	94.4%	*94.7*%	77.8%	100%	*100*%	100%
Accuracy	90.0%	*92.5*%	75.0%	97.5%	*97.5*%	90.0%
*pvalue*	*<0.001*	*<0.001*	*<0.001*	*<0.001*	*<0.001*	*<0.001*
Cutoff value	<3.14 kPa	*<7.28* kPa	<9.14 kPa	<5.32 kPa	*<10.47* kPa	<15.16 kPa

**Table 5:** RTE results: SR values.

	Min SR	Max SR	Mean SR
Parathyroid/thyroid SR	0.01	5.82	1.46 ± 1.45
Parathyroid/muscle SR	0.5	3.5	1.79 ± 0.90

## Data Availability

The data that support the findings of this study are available from the corresponding author upon reasonable request.

## Abbreviations

PTH: Parathyroid hormone
SR: Strain ratio
PHPT: Primary hyperparathyroidism
VTIQ: Virtual touch imaging quantification
ARFI: Acoustic radiation force impulse
MIBI: Technetium sestamibi scintigraphy
RTE: Real-time elastography
SD: Standard deviation
SWV: Shear wave velocity
2D-SWE: 2 dimensional shear wave elastography
US: Ultrasonography
Sn: Sensibility
ROI: Region of interest
Sp: Specificity
EI: Elasticity index
SWE: Shear wave elastography.
